# Comparative Nutritional and Healthy Values of Macro- and Microelements in Edible and Non-Edible Tissues of Raw and Processed Common Octopus (*Octopus vulgaris*)

**DOI:** 10.3390/foods14132210

**Published:** 2025-06-23

**Authors:** Ricardo Prego, Antonio Cobelo-García, Susana Calvo, Santiago P. Aubourg

**Affiliations:** 1 Department of Oceanography, Marine Research Institute (CSIC), c/E. Cabello 6, 36208 Vigo, Spain; acobelo@iim.csic.es (A.C.-G.); scalvo@iim.csic.es (S.C.); 2 Department of Food Technology, Marine Research Institute (CSIC), c/E. Cabello 6, 36208 Vigo, Spain

**Keywords:** common octopus, arm, mantle, viscera, cooking, frozen storage, macroelements, microelements, nutrition, sustainability

## Abstract

The macroelement (Na, Mg, P, S, K, and Ca) and microelement (Mn, Fe, Co, Cu, Zn, As, Cd, Sn, Ba, and Pb) composition of edible (arm and mantle) and non-edible (viscera) tissues of octopus (*Octopus vulgaris*) was studied. Three different size groups were considered separately (1–2, 2–3, and 3–4 kg per specimen). Additionally, the effect of cooking processing (40 min at 90 °C) and frozen storage (4 months at –18 °C) was determined. All raw tissues depicted the following increasing sequence for the macroelement content (*p* < 0.05): Ca < Mg < P ≈ K < Na ≈ S; regarding microelements, the raw viscera tissue showed a higher level (*p* < 0.05) than the counterpart edible tissues. The cooking process led to a general decrease in macroelement values (*p* < 0.05) in arm and mantle tissues; for microelements, no effect (*p* > 0.05) was observed for Co, Mn, and Sn content, but an average increase was obtained for Cd, Cu, and Pb values. The frozen storage did not lead to element content changes in the arm tissue (*p* > 0.05); in contrast, general content increases and decreases were detected for mantle and viscera, respectively. In spite of level changes detected, this study proves that viscera, a common waste of commercial processing, can be considered a valuable source of essential elements.

## 1. Introduction

Seafood provides a high content of important constituents for the human diet [1]. As living in a mineral-rich medium, marine organisms accumulate macro- and microelements from the diet and the marine medium and incorporate them in their tissues and organs [2,3,4]. Notably, a wide range of such elements are considered essential for biological processes and can be found abundantly in different kinds of tissues of marine species [5,6,7]. Among trace elements, Cu, Se, Mn, and Zn are contained in enzymes which protect cells against oxidant stress and therefore may be considered biological antioxidants [8].

However, the presence of toxic trace elements has led to some health risks in commercially available seafood [9,10,11]. Thus, marine species have been reported to be valuable bioindicators of trace element contamination in the marine environment because they occupy different trophic levels and can exhibit large bioaccumulation factors [12,13,14]. Both for essential and toxic elements, previous research has proved that their concentration in the different tissues of marine species may be influenced not only by external factors (food source and environment) but also by anatomical and physiological aspects [15,16,17].

Marine species are known to be especially prone to deterioration during the processing technologies employed commonly for commercial purposes. Thus, heat treatment [18,19] or frozen storage [20,21] may lead to a wide range of different damage pathways, such as nutrient degradation, oxidation of vitamins and lipids, muscle toughening, microstructural changes, protein denaturation, or constituent hydrolysis. As a result, sensory and nutritional losses can occur, leading to a remarkable decrease in the consumer’s acceptance [22,23,24]. Such deteriorative events can be very important for the macro- and microelement presence in seafood. Element release resulting from breakdown and denaturation of constituents, especially the protein fraction, is likely to lead to a remarkable loss of the element presence in the resulting seafood and provoke a notable loss of the nutritional value [25,26,27]. Compared to the number of studies focused on protein and lipid changes resulting from seafood processing, those regarding macro- and microelement values can be considered scarce [28,29].

As a result of the processing of marine species, the seafood industry generates a great quantity of discards and waste. Such substrates are known to include a wide variety of healthy and nutritional constituents (lipids, proteins, minerals, and vitamins) [30,31] but can also be considered a relevant environmental concern of coastline areas [32,33]. Therefore, great efforts are nowadays carried out for their recovery in agreement with current commitments for environmental sustainability and circular economy. Regarding the profitable employment of such by-products by nutraceutical, pharmaceutical, and cosmeceutical industries, most stress has been accorded to protein, vitamin, and lipid fractions [34,35]; in contrast, the number of studies focused on the use of minerals can be considered very short [8,11,36].

Octopus species constitute highly nutritional seafood that are commercialized in a great variety of products and show great acceptability from consumers [37,38]. Edible parts of the octopus body have deserved a wide range of studies focused on their nutritional value [7,17,39,40] and quality changes during processing [41,42]. In contrast, non-edible tissues have been scarcely studied [12,29]. The present research provides a comparative study of the macro- and microelement content in edible (mantle and arm) and non-edible (viscera) tissues of common octopus (*Octopus vulgaris*). Additionally, the effect of cooking processing and frozen storage was also studied.

## 2. Materials and Methods

### 2.1. Octopus Sampling and Processing

Specimens corresponding to common octopus (*O. vulgaris*), fished near the Galician coast (NW Iberian Peninsula, April 2023), were supplied by Frigoríficos Rosa de los Vientos S. L. (Marín, Pontevedra, Spain). Three different groups were considered separately based on the individual weight of specimens: Group I (1–2 kg), Group II (2–3 kg), and Group III (3–4 kg). With the aim of carrying out this study, 24 specimens of each group were considered. Within each group, separation of mantle, arm, and total viscera was carried out.

Regarding the mantle tissue, one part of the ‘raw mantle’ (six specimens) was directly subjected to analysis, i.e., three independent batches (*n* = 3) with mantle corresponding to two specimens per batch. Another part (six specimens) was subjected to freezing (48 h at –40 °C; Sanyo Ultralow MDF-U53W, Moriguchi, Osaka, Japan) followed by frozen storage (4 months at –18 °C; Radiber, Barcelona, Spain); after this period, ‘frozen mantle’ samples were subjected to thawing (overnight refrigerated storage at 4 °C) and subsequent analysis (three independent batches, *n* = 3, with frozen mantle corresponding to two specimens per batch). The remaining twelve samples of mantle tissue were subjected to the cooking process (40 min at 90 °C; Presoclave II 75L, JP Selecta, Barcelona, Spain); after cooking, one half, i.e., six specimens of ‘cooked mantle’ samples, were subjected directly to analysis. The other half of the cooked mantle samples (six specimens) was subjected to freezing (48 h at –40 °C), followed by frozen storage (4 months at –18 °C) (three independent batches, *n* = 3, with frozen mantle corresponding to two specimens per batch); after this period, the ‘cooked frozen mantle’ was subjected to thawing (overnight storage at 4 °C) and subsequent analysis.

For the arm tissue, the sampling procedure carried out was the same as for the mantle tissue. Consequently, the following arm samples were obtained and named ‘raw arm’, ‘frozen arm’, ‘cooked arm’, and ‘cooked frozen arm’.

With respect to viscera, ‘raw viscera’ tissues of twelve specimens were directly subjected to analysis. The remaining raw viscera samples, corresponding to twelve specimens, were subjected to freezing (48 h at –40 °C) followed by frozen storage (4 months at –18 °C) (three independent batches, *n* = 3, with frozen viscera corresponding to four specimens per batch); after this period, the ‘frozen viscera’ samples were subjected to thawing (overnight storage at 4 °C) and subsequent analysis. According to industrial practice, the viscera tissue was not subjected to cooking processing.

### 2.2. Determination of the Moisture Content

Moisture was determined as the weight difference (1–2 g tissue) before and after 4 h at 105 °C according to official method 950.46B [43]. Results were calculated as g·100 g^−1^ tissue.

### 2.3. Chemical Element Determination

Contents of six macroelements (Ca, K, Mg, Na, P, and S) and ten microelements (As, Ba, Cd, Co, Cu, Fe, Mn, Pb, Sn, and Zn) were determined in the octopus samples according to a procedure based on US-EPA 3050B [44] and in agreement with previous research [45,46]. About 1.5 g of sample (wet weight) was put into a Teflon digestion flask with 4 mL of 69% nitric acid (TMA) Hiperpur and 4 mL of H_2_O_2_ (for ultra-trace analysis). Samples plus six blanks and six certified reference materials (around 0.3 g each) were digested in a microwave oven (Mars-Xpress CEM Corp., Matthews, NC, USA). After complete digestion, solutions were transferred to 50 mL flasks, filling that volume with Milli-Q water. Handling of samples was carried out inside a clean ISO 5 laminar flow cabinet (Cruma 670 FL, Barcelona, Spain). In the digested samples, the determination of macro- and microelement concentrations was carried out using a 7900 Agilent ICP-MS operated in the He mode for all elements with the exception of Cd, Sn, Ba and Pb (no-gas/standard mode was used). An inert sample introduction system for the ICP-MS determination was employed, consisting of a PFA nebulizer and spray chamber (operated at 2 °C), a sapphire injector and Pt cones. The instrumental parameters were daily optimized before measurements in order to obtain the highest intensity and signal stability while maintaining low oxide (^140^Ce^16^O^+^/^140^Ce^+^ < 1%) and double charge (^140^Ce^2+^/^140^Ce^+^ < 3%) formation. The analytical determination was attained using external calibration with a multi-elemental standard. Germanium, Rh, and Ir were used as internal standards in order to correct for instrumental drift and matrix effects. Blanks (1 per 10 samples) were prepared using the digestion procedure described above; results presented here are therefore blank-corrected. Accuracy of the analytical procedures was ensured using DORM-5 (Fish Protein Certified Reference Material) from NRC (National Research Council Canada), which was digested and analysed in the same batch as the samples showed good agreement with the certified concentrations (Table 1). The limits of detection, expressed as 3·SD-blank, were 0.0004 mg Ca·g^−1^, 0.002 mg K·g^−1^, 0.0004 mg Mg·g^−1^, 0.005 mg Na·g^−1^, 0.002 mg P·g^−1^, 0.035 mg S·g^−1^, 0.05 mg As·kg^−1^, 0.0001 mg Ba·kg^−1^, 0.00005 mg Cd·kg^−1^, 0.0007 mg Co·kg^−1^, 0.007 mg Cu·kg^−1^, 0.05 mg Fe·kg^−1^, 0.0025 mg Mn·kg^−1^, 0.0001 mg Pb·kg^−1^, 0.0001 mg Sn·kg^−1^, and 0.08 mg Zn·kg^−1^.

Content of macro- and microelements in the different tissue samples was calculated as g·kg^−1^ and mg·kg^−1^ wet tissue, respectively.

### 2.4. Statistical Analysis

This study was carried out in triplicate (*n* = 3). For each kind of sample (specimen size, tissue, and raw/processed), three independent batches were employed. In the case of arm and mantle, each sample analysed was composed of tissues corresponding to two different specimens. For viscera, each sample analysed was composed of tissues corresponding to four different specimens.

Data obtained from the analysis of the chemical elements were subjected to the ANOVA method. For it, one-way ANOVA was applied to investigate differences resulting from the following factors: tissue, cooking process, and freezing and frozen storage. Statistical comparisons were conducted via PASW Statistics 18 Software for Windows (Statistica version 6.0, 2002; Statsoft Inc., Tulsa, OK, USA). Comparison of means was performed using the least-squares difference (LSD) test. The 95% confidence intervals of each element parameter were calculated; for it, the standard deviation of each sample and the number of replicates were considered.

## 3. Results

### 3.1. Moisture Determination

Moisture showed to be the most abundant constituent, being included in all cases in the 73–82 g·100 g^−1^ range. A lower (*p* < 0.05) moisture value was detected in raw viscera (73–75 g·100 g^−1^ range) when compared to raw arm and mantle tissues (79–81 and 81–82 g·100 g^−1^ ranges, respectively). As a result of the frozen storage, arm, mantle, and viscera tissues did not reflect remarkable differences (*p* > 0.05) in moisture value (77–80, 81–82, and 71–78 g·100 g^−1^ ranges, respectively). In contrast, the cooking processing led to losses of moisture presence in arm and mantle tissues (76–77 and 78–79 g·100 g^−1^ ranges, respectively).

### 3.2. Comparative Content of Macroelements in the Different Raw Tissues

**Figure 1:**
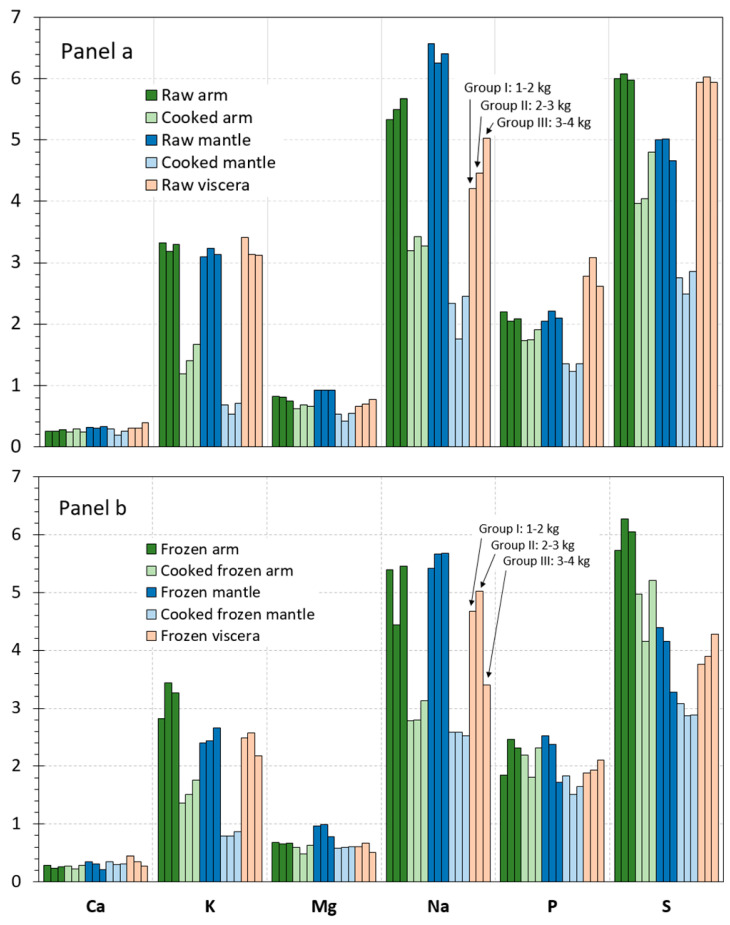
Macroelement contents (g·kg^−1^ wet tissue) in raw and processed tissues of octopus. Panel (**a**): raw and cooked samples; Panel (**b**): frozen and cooked frozen samples. Three size groups were considered according to the weight sizes of octopus specimens, i.e., Group I (1–2 kg), Group II (2–3 kg), and Group III (3–4 kg).

The contents obtained for macroelements (Ca, K, Mg, Na, P, and S) in each of the different size groups of raw octopus tissues are displayed in Figure 1a. In it, each bar represents a weight group, whose height is the result of the average value of a three-sample analysis (*n* = 3), and the colour code serves to identify the different kinds of tissues. Globally, macroelement contents ranged from 0.2 g Ca·kg^−1^ in arm tissue to 6.6 g Na·kg^−1^ in mantle tissue. In general terms, the following increasing sequence for the element content was detected in the three tissues: Ca < Mg < P ≈ K < Na ≈ S.

Similar levels were detected among tissues for the Ca and K presence (Figure 1). For such elements, average values in all raw tissues were included in the 0.2–0.4 and 3.1–3.4 g·kg^−1^ ranges, respectively. Regarding Mg, the highest average value was detected in the mantle tissue (ca. 0.9 g·kg^−1^), values corresponding to the two other tissues being included in the 0.7–0.8 g·kg^−1^ range. In the case of the Na content, the following decreasing sequence was observed for the different tissues: mantle (6.3–6.5 g·kg^−1^) > arm (5.3–5.6 g·kg^−1^) > viscera (4.2–5.0 g·kg^−1^). The analysis of the three size groups showed a higher P content in viscera (2.6–3.1 g·kg^−1^) than in arm and mantle tissues (2.0–2.2 g·kg^−1^). Finally, the lowest S value was observed in the mantle tissue (4.7–5.0 g·kg^−1^); arm and viscera tissues showed levels included in the 5.9–6.1 g·kg^−1^ range.

For each macroelement and for each octopus tissue, some differences could be detected as a result of the weight size. However, such range values can be considered relatively tight. Thus, it could be concluded that the effect of the octopus size was negligible. Therefore, data obtained for the three sizes were pooled together, and the resulting data were analysed statistically. Results obtained after this data treatment are depicted in Table 2.

As a result, some differences could be outlined by comparison of the three raw tissues. Thus, the viscera tissue revealed higher (*p* < 0.05) *p* values than the other tissues. The mantle tissue showed higher (*p* < 0.05) Mg levels but lower (*p* < 0.05) S contents than the counterpart tissues. The arm tissue showed the lowest (*p* < 0.05) presence of Ca. Regarding the Na content, the following decreasing (*p* < 0.05) sequence was detected for the different tissues: viscera < arm < mantle. Finally, no differences (*p* > 0.05) among tissues were proved for the K presence.

### 3.3. Comparative Content of Microelements in the Different Raw Tissues

**Figure 2:**
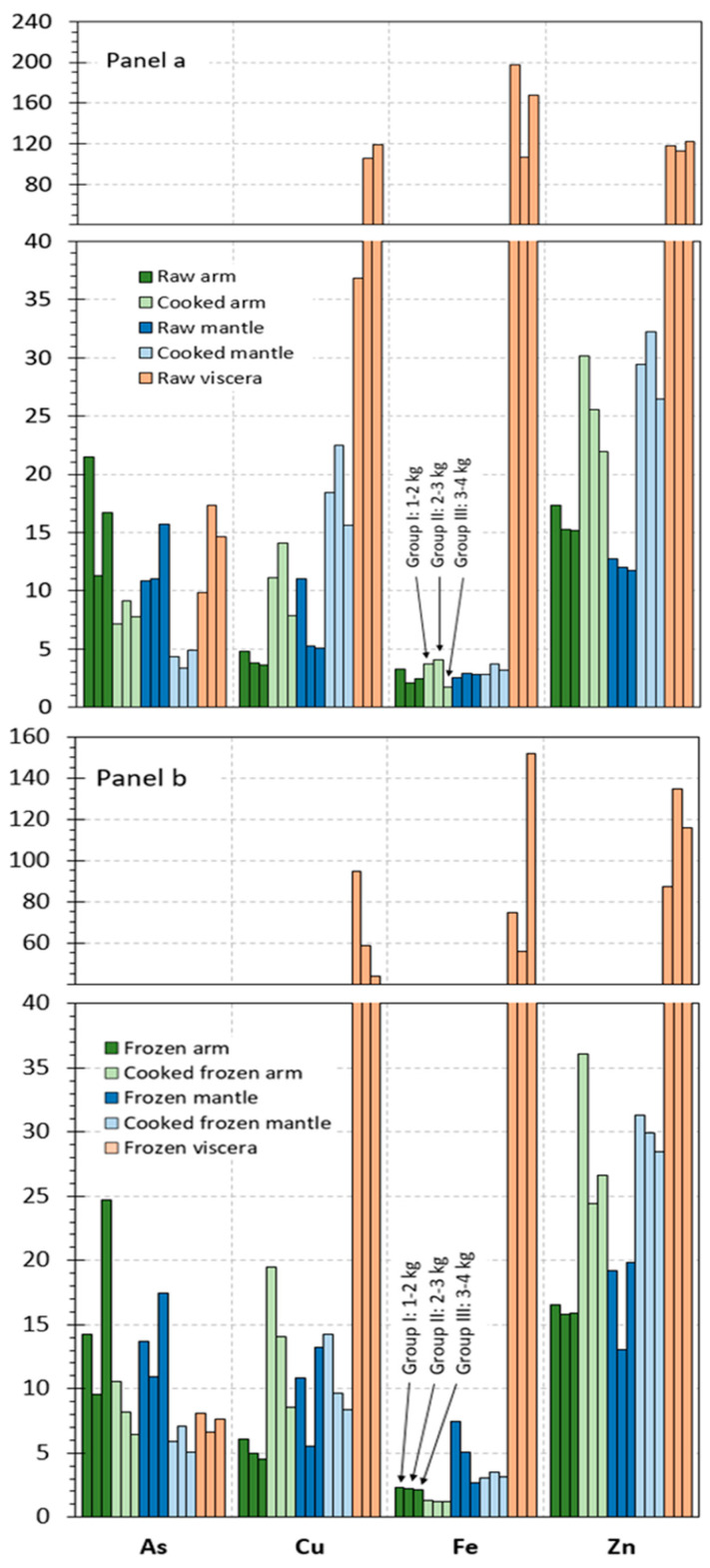
Microelement (As, Cu, Fe, and Zn) contents (mg·kg^−1^ wet tissue) in raw and processed tissues of octopus. Panel (**a**): raw and cooked samples and Panel (**b**): frozen and cooked frozen samples. Three weight sizes of octopus specimens, as expressed in Figure 1.

**Figure 3:**
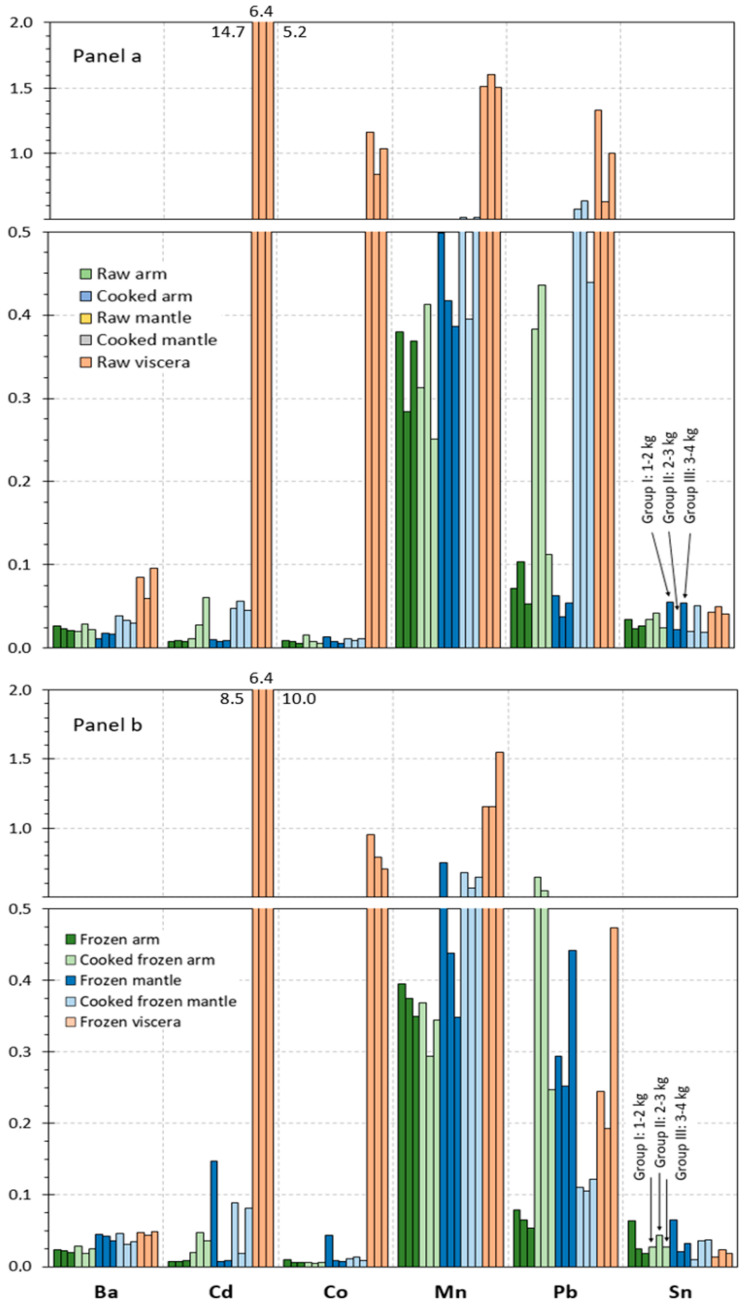
Microelement (Ba, Cd, Co, Mn, Pb, and Sn) contents (mg·kg^−1^ wet tissue) in raw and processed tissues of octopus. Panel (**a**): raw and cooked samples and Panel (**b**): frozen and cooked frozen samples. Three weight sizes of octopus specimens as expressed in Figure 1.

The contents obtained for microelements in the different raw tissues corresponding to the different size groups of octopus specimens are represented in Figure 2a and Figure 3a. Results on microelements are distributed into Figure 2 and Figure 3, according to the content ranges obtained for the different elements. Thus, Figure 2a includes those elements whose values are included in the 2–200 mg·kg^−1^ range (As, Cu, Fe, and Zn), and Figure 3a depicts elements whose presence is included in the 0–2 mg·kg^−1^ range (Ba, Cd, Co, Mn, Pb, and Sn).

In most cases (Cu, Fe, Zn, Ba, Cd, Co, Mn, Pb, and Sn), the highest average values in all size groups were detected in the viscera tissue. Thus, values included in the 37–119, 107–198, 112–122, 0.06–0.10, 5.2–14.8, 0.8–1.2, 1.5–1.6, and 0.6–1.3 g·kg^−1^ ranges, respectively, were obtained in raw viscera. Similar values were observed in the arm as in the mantle for Fe (2.1–3.3 g·kg^−1^), Cd (0.007–0.010 g·kg^−1^), and Co (0.006–0.013 g·kg^−1^). Higher ranges were detected in the mantle tissue for Cu (5.1–11.0 g·kg^−1^) and Mn (0.4–0.5 g·kg^−1^) than in the arm tissue. In contrast, the arm tissue depicted higher ranges for Zn (15.1–17.3 g·kg^−1^), Ba (0.02–0.03 g·kg^−1^), and Pb (0.07–0.10 g·kg^−1^) than the mantle tissue. Finally, similar values in all tissues were observed for As (9.8–21.5 g·kg^−1^).

Although raw tissues provided some differences as a result of the size group, differences found did not lead to general trends. Therefore, and as in the case of the macroelement analysis, data corresponding to the different specimen sizes were pooled together in order to accomplish the statistical analysis. The corresponding results obtained after carrying out such analysis are shown in Table 3 and Table 4.

The statistical analysis depicted a higher (*p* < 0.05) content in viscera tissue for Cd, Co, Cu, Fe, Pb, and Mn elements when compared to the two edible tissues. Additionally, the following increasing (*p* < 0.05) sequence was detected for the Ba and Zn values in the different tissues: mantle < arm < viscera. In contrast, no effect (*p* > 0.05) of the raw tissue considered was observed for the As presence.

### 3.4. Effect of Cooking Processing on Element Content

#### 3.4.1. Macroelement Content

Values detected for the macroelement content in arm and mantle tissues corresponding to cooked and cooked frozen samples are provided in Figure 1a,b. In such figures, values obtained for each of the size groups of octopus specimens are shown. In order to determine the effect of cooking, a comparison to raw samples (Figure 1a) is carried out.

A relevant decrease value was detected in all size groups for most elements as a result of cooking in both cooked arm and mantle tissues. Samples of all groups depicted decreasing tendencies for K, Mg, Na, P, and S contents after the thermal treatment; in contrast, Ca did not undergo level differences after cooking.

On the basis that differences among the three size groups were scarce in arm and mantle tissues, data corresponding to the different size groups were pooled together and the resulting data analysed statistically. The corresponding results obtained after carrying out such statistical treatment are shown in Table 2.

As a result, a remarkable decrease (*p* < 0.05) of the K, Na, P, and S values was proved in cooked arm and mantle tissues when compared to the counterpart raw tissues. Average values of Mg also provided a decreasing tendency with cooking processing; however, differences were only significant (*p* < 0.05) in the case of the cooked and cooked frozen mantle tissues. In contrast, no effect (*p* > 0.05) was proved for Ca values in arm and mantle tissues as a result of the thermal treatment.

#### 3.4.2. Microelement Content

Values detected for the microelement content in cooked arm and mantle tissues are provided in Figure 2a and Figure 3a. In such figures, values obtained for each of the size groups of octopus specimens are depicted.

An increasing tendency was observed in cooked arm and mantle for Cu, Zn, Cd, and Pb contents; in contrast, the As value underwent a decrease as a result of the thermal treatment. Remarkably, the content of elements such as Fe, Ba, Co, Mn, and Sn did not show differences in cooked arm and mantle tissues when compared to the counterpart raw tissues.

As for macroelement analysis, data obtained in the different size groups were pooled together in order to evaluate the results obtained statistically. Differences in the microelement content resulting from the cooking treatment of raw arm and mantle are depicted in Table 3 and Table 4.

Thus, no effect (*p* > 0.05) of the cooking process was proved for the Co, Mn, and Sn presence in the arm and mantle tissues corresponding to cooked samples. In contrast, increasing and decreasing (*p* < 0.05) values were detected for Zn and As, respectively, in cooked samples corresponding to both tissues. An average increase in the Cd, Cu, Fe, and Pb presence was observed in most cooked samples; differences with the counterpart raw samples were found significant (*p* < 0.05) in cooked arm tissue. Regarding the Ba value, no effect (*p* > 0.05) of the cooking treatment was concluded, except for the cooked mantle tissue, which showed an increasing (*p* < 0.05) content by comparison to its counterpart raw sample.

### 3.5. Effect of Freezing and Frozen Storage on Element Content

#### 3.5.1. Macroelement Content

Values detected for the macroelement content in frozen and cooked frozen tissues are provided in Figure 1b. In such a figure, values obtained for each of the size groups of octopus specimens are represented. Comparison to raw and cooked samples, respectively, is carried out to determine the effect of freezing and frozen storage.

Comparison of values included in Panels a and b of Figure 1 shows that the content of the macroelements in the frozen and cooked frozen samples corresponding to the arm tissue underwent very scarce differences as a result of the freezing and frozen storage process. In contrast, the raw viscera depicted a general loss of macroelements after the frozen storage in all size groups. In the case of the mantle tissue, a general trend could not be concluded; thus, a negligible effect could be outlined for Ca and Mg, and a decreasing tendency for the K and S values could be observed.

On the basis that differences among the three size groups considered in this study were found to be scarce, data were pooled together as in previous parts of the manuscript. Thus, the statistical analysis of changes produced in raw and cooked samples as a result of freezing and frozen storage was analysed statistically. Differences resulting from the macroelement content in frozen and cooked frozen tissues are depicted in Table 2.

In most cases, the frozen and cooked frozen arm tissue did not provide significant differences (*p* > 0.05) for the macroelement content; remarkably, the only significant difference (*p* < 0.05) found was the Na content decrease in cooked frozen samples when compared to the counterpart cooked samples. A general average decrease was detected in frozen viscera for all macroelements considered in this study; differences were found significant (*p* < 0.05) for K, Mg, P, and S values. Decreased (*p* < 0.05) K, Na, and S values were observed in frozen and cooked frozen mantle samples; in contrast, increased *p* values (*p* < 0.05) were detected in frozen and cooked frozen mantle tissues.

#### 3.5.2. Microelement Content

Data obtained for the microelement content in frozen and cooked frozen tissues are depicted in Figure 2b and Figure 3b. In such figures, values obtained for each of the size groups of octopus specimens are represented. As for macroelements, comparison to raw and cooked samples is carried out to determine the effect of freezing and frozen storage.

A comparison of results obtained indicated different tendencies for the three tissues. Thus, the arm tissue, both for frozen and cooked frozen samples, showed negligible differences as a result of the freezing and frozen storage. In contrast, the results obtained for all size groups of octopus specimens indicated a general content decrease in most microelements under study for the viscera tissue. Finally, an increasing tendency could be observed for the mantle tissue content in general, especially in the case of samples without previous cooking processing.

On the basis that differences among the three size groups considered in this study were found scarce, data regarding microelement content were pooled together as in previous parts of the manuscript. Thus, the statistical analysis of changes produced as a result of freezing and frozen storage is expressed in Table 3 and Table 4.

In general, the arm tissue did not undergo changes (*p* > 0.05) for the microelement content; however, a decreasing Fe presence (*p* < 0.05) in cooked frozen samples was observed when compared to the counterpart cooked samples. For the mantle tissue, increases in average contents for most elements were detected; differences were found significant (*p* < 0.05) for Ba, Cd, Fe, Pb, and Zn (frozen samples) and for As and Mn (cooked frozen samples). In contrast to this tendency, the cooked frozen mantle indicated decreased values (*p* < 0.05) for Cu and Pb. Regarding the viscera tissue, a general average decrease for all elements could be observed; such decreases were found significant (*p* < 0.05) for As, Ba, Pb, Fe, Mn, Sn, and Zn.

## 4. Discussion

### 4.1. Comparative Content of Elements in the Different Raw Tissues

Concentrations of elements in raw cephalopod tissues and marine species in general can be influenced by a wide number of factors such as seasonal and biological differences, nourishment source, and environmental conditions [29,47,48,49].

In spite of differences among raw tissues in the content of essential elements, valuable levels have been obtained in all tissues in agreement with other kinds of marine substrates [2,8,50]. This result can be especially important for the viscera tissue, a substrate commonly discarded after the commercial processing. Notably, the viscera tissue showed higher (*p* < 0.05) values than the edible tissues for P, Co, Cu, Fe, Mn, and Zn; in contrast, a lower (*p* < 0.05) Na presence was detected.

Regarding toxic elements, values detected for Ba, Cd, and Pb in edible tissues were notably lower (*p* < 0.05) than in viscera. At this point, the European Commission regulation EU 2023/915 of Commission 25th April 2023 [51] set maximum levels for certain contaminants in foodstuffs, which for Pb was 0.3 mg·kg^−1^ (eviscerated cephalopod), and for Cd was 1 mg·kg^−1^ (eviscerated cephalopod). Additionally, the EFSA set the tolerable weekly intake for Cd at 2.5 kg/week/kg of body weight [52]. Therefore, and before carrying out any attempt for the commercialization of the current viscera tissue, such limitations ought to be taken into account. Concerning the As content, no limit recommendation is actually available for total As in seafood [53].

Previous research regarding the mineral composition of octopus tissues has focused on edible tissues; in contrast, composition reports on non-edible tissues can be considered scarce. In agreement with previous comments, great differences were detected in previous studies for the current octopus (*O. vulgaris*) species on the basis of the site, location, and tissue taken into account.

Thus, heavy metal concentrations were determined in various tissues of specimens collected from three different contaminated sites in front of Alexandria (Egypt) [12]. As a result, higher levels than in the present case were detected in the mantle for Cd, Co, Fe, Mn, and Pb; in contrast, mantle contents were found similar for Cu and Zn. Compared to the current raw viscera values, Nessim and Riad [12] found higher levels for Cd, Co, Cu, and Pb in the digestive gland; however, values were similar for Fe and Mn.

Later on, Lourenço et al. [7] analysed the element composition of the edible part of common octopus obtained in Portuguese continental waters from May to September 2004–2005. As a result, lower contents than in the present study (raw arm and mantle tissues) were detected for all macroelements (Ca, K, Mg, Na, P, and S). Regarding microelements, levels for Cu, Fe, Mn, and Zn were found to be more similar than in both current edible tissues; in contrast, higher and lower levels were detected for Cd and Pb, respectively, when compared to the current research.

A wide study of the element composition of different tissues of common octopus captured in three different locations of the W-Mediterranean Sea was carried out by Arechavala-López et al. [17]. Regarding the edible tissue (mantle), the authors detected higher levels of macroelements (Ca, Na, and K) and microelements (Mn, Fe, Co, Cu, Zn, As, Cd, Ba, and Pb) than in the present study for raw mantle; in contrast, a lower content of Mn was detected than in the current study. Additionally, higher levels for all macro- and microelements analysed were detected in the digestive gland when compared to the present raw viscera values [17].

Recently, the accumulation levels of different microelements in different tissues were analysed in octopus specimens captured in the Marmara, Aegean, and Mediterranean Seas [29]. Regarding the mantle tissue, the authors found higher levels than in the present raw mantel for Co, Pb, Fe, and Zn; in contrast, values for Mn and Cu were lower than in the current study. Compared to the present raw viscera values, Duysak et al. [29] reported higher levels in the digestive gland for Co, Pb, Mn, Fe, Cu, and Zn; in contrast, a lower presence was observed for Cd.

### 4.2. Effect of Cooking Processing on Element Content

Except for Ca, the present results have shown a general decrease in the macroelement content in arm and mantle tissues as a result of the cooking process. In contrast, the Ca value did not provide any difference in any of the edible tissues. In the case of microelements, a general trend could not be concluded. Thus, no effect was observed for the Co, Mn, and Sn values; in contrast, an average increase was obtained for the presence of Cd, Cu, and Pb.

In order to explain such differences in tendencies, it can be argued that changes can be influenced by several factors. On one side, the cooking process would lead to a partial damage of constituents (especially in the protein and lipid fractions), consisting of breakdown and content loss [18,19,54]. Notably, the release of prooxidant elements such as non-haem Fe from haem-Fe complexes as a result of protein denaturation may have important consequences in the rancidity stability of the marine substrate [25,55]. As a consequence of protein and lipid damage and breakdown, losses of such constituents would lead to relative content increases in other constituents in the marine substrate, such as macro- and microelements. Additionally, a loss of moisture content has been reported to be produced during cooking processing and thermal treatment in general in seafood [19,25]. Thus, a partial moisture loss was described in Section 3.1. for arm and mantle tissues as a result of the cooking process.

On the other side, modifications of the different main constituents resulting from cooking processing would lead to the breakdown of the binding of elements to such constituents. Among the different constituent modifications, protein denaturation can be of special significance for element content in marine tissues. As a result, liquor losses would lead to partial losses of such elements into the surrounding medium. Related to such losses, the kind of binding of elements to other constituents would play a decisive role and show a great dependence on their chemical characteristics [2,4,8]. Thus, alkali (Na and K) and alkali earth (Mg and Ca) elements have been shown to be present in the cellular medium as chlorides, sulphates, or organic salts (citrates, lactates, or pyruvates). In contrast, transition metals (Fe, Cu, etc.) and non-positive elements (S, P, etc.) have been shown to be strongly bound to other constituents and give rise to a wide number of functional molecules.

Some authors have reported that element contents can vary in accordance with cooking time and method [56]. Another source of variation is that mineral matter interacts with other nutrients, such as proteins, and can alter the bioavailability of some elements more than others [57]; this effect has been reported to be especially important in divalent elements such as Ca, Mg, Fe, and Zn [58], due to reduced protein quality hindering the ability to form complexes with these elements [59].

Previous research accounts for information regarding changes of the element presence in edible tissues of common octopus (*O. vulgaris*) as a result of cooking or thermal treatment in general. A general increase (mg·kg^−1^ wet tissue) in chemical macroelements (Na, K, Mg, and Ca) and microelements (Mn, Cu, Fe, Zn, Pb, Cd, and Hg) was detected in common octopus (*O. vulgaris*) as a result of the frying process in sunflower oil [60]. Alves et al. [61] proved that steaming affected differentially the element content in edible tissues of octopus; after processing, Hg, Zn, Se, and Cu levels showed an increase; As, Cd, and Fe values underwent a decrease; and Mn, Sr, and I did not reveal a content variation. Oliveira et al. [41] analysed the effect of the boiling process on the element content in common octopus. After the heating process, edible tissues showed an increase in P, Cu, Zn, and Hg levels; a decrease in Na and I values; and practically no changes in Mg, K, Se, Cd, and Pb levels. After sous-vide cooking, K, Mg, Na, and P contents of octopus decreased; those of Ca and Zn increased; and values for Fe were not affected [42].

### 4.3. Effect of Freezing and Frozen Storage on Element Content

In the present study, the response to the freezing and frozen storage processing has proved to be relatively different according to the tissue considered. Both for the macro- and microelement contents, the arm tissue provided very scarce differences. In contrast, a general decrease could be concluded for the viscera tissue. Finally, the mantle tissue showed general increases for the microelement content but general decreases for the macroelement presence.

As in the case of considering the effect of the cooking process, changes detected can be the result of different factors. On the one side, the freezing and frozen storage process would lead to partial damage of constituents, consisting of breakdown and content loss. This effect would be of special significance as a result of protein denaturation [20,23] and lipid oxidation by endogenous enzyme action (i.e., peroxidases, lipoxygenases, oxidases, etc.) [22,62]. Release of pro-oxidant elements such as non-haem Fe from haem-Fe complexes as a result of protein denaturation may have important consequences on rancidity stability [25,55]. Consequently, losses on such constituents would lead to relative content increases in other constituents in the marine substrate, such as macro- and microelements. In contrast to the cooking process, the freezing and frozen storage did not lead to changes of the moisture value (Section 3.1). Therefore, no effect of the relative presence of this constituent would be expected to occur on the element content in the different tissues.

On the other side, and as previously mentioned for the cooking process, modifications of the main constituents can lead to the breakdown of the binding of microelements to other constituents. Thus, protein denaturation resulting from freezing and frozen storage can be of great significance, as liquor losses would lead to partial losses of elements into the surrounding medium. In this sense, the kind of binding of elements to other constituents, as well as the more or less hydrophilic/lipophilic behaviour of molecules they are integrated in, ought to be taken into account [2,4,8]. Thus, hydrophilic molecules will be likely to be lost during processing steps such as thawing. As previously mentioned, alkali and alkali earth elements are especially present in the cellular medium, while transition metals and non-positive elements are likely to be bound to other constituents.

Regarding the effect of freezing and frozen storage on changes in macro- and microelement contents in octopus and seafood in general, previous research can be considered scarce in the case of edible tissues; for non-edible tissues of cephalopod species and seafood in general, previous related research can be considered negligible.

Thus, the content of non-haem Fe increased during frozen storage of cod (*Gadus morhua*) and mackerel (*Scomber scombrus*) due to haem breakdown [63]; on the basis that storage above –14 °C was more deleterious than a lower temperature (–20 or –40 °C), the authors concluded that a higher frozen storage temperature may lead to a slightly faster decrease in non-haem Fe. Furthermore, Pourashouri et al. [64] showed an increase in the non-haem Fe level as a result of the Fe release from haem-Fe complexes during the frozen period (6 months at –18 °C) of Wels catfish (*Silurus glanis*); this change was explained according to the oxidative cleavage of the porphyrin ring [55]. Karl et al. [65] proved a marked reduction in I content in different kinds of fish after deep-freezing and thawing. A previous 6–month frozen period (–18 °C) led to a general content decrease in essential element (K, Mg, Ca, Mn, Fe, Se, P, and S) content in brine-canned mackerel (*Scomber colias*) [45]. In a subsequent study, the same authors analysed the effect of the previous frozen conditions on the content of essential elements when a longer storage period (15 months) was employed at the same storage temperature (–18 °C) in brine-canned mackerel (*S. colias*) [27]; as a result, an increased frozen storage time led to an increase in Ca and Mn contents but produced a decrease in the K value. Recently, a previous frozen storage (6 months at –18 °C) led to an increased presence of K, Ca, Cu, and Mn in canned Atlantic mackerel (*S. scombrus*) [46]; in contrast, decreased values were observed for P and S.

## 5. Conclusions

The macro- and microelement composition of edible and non-edible tissues of raw and processed samples of common octopus (*O. vulgaris*) was studied. Although three size specimens were considered separately, substantial differences in element composition were not detected. Consequently, size groups were pooled together in order to achieve the statistical analysis of data obtained. In spite of content variations as a result of tissue or processing, present results have shown a valuable content of essential and nutritional elements in all raw and processed tissues in agreement with other kinds of marine substrates. This result can be especially important for the current viscera tissue, a waste substrate commonly discarded after the commercial processing. Regarding toxic elements, the highest levels for Ba, Cd, and Pb were detected in the viscera tissue. Therefore, and before carrying out any attempt for the commercialization of the viscera tissue, current international commitments ought to be taken into account.

Previous reports on element contents in processed seafood can be considered scarce in general when compared to other marine constituents such as the protein and lipid fractions. To the best of our knowledge, this study provides a first comparative study focused on the content of macro- and microelements of raw and processed edible and non-edible tissues of common octopus, a highly appreciated marine species. This novel approach meets the current global interest in the search for natural sources of bioactive compounds, such as essential elements from raw and processed seafood, to be employed for human nutrition, as well as for nutraceutical, pharmaceutical, and cosmeceutical industries. Besides the added value that by-products (i.e., the current viscera tissue) may represent as natural sources, there is also the ecological upside of reducing seafood industry waste. In this sense, repurposing these kinds of bioresources will contribute to a more sustainable use of seafood.

In agreement with the influence that external (i.e., catching season and location) and internal (i.e., gender, maturity degree, and size) factors may have on the element distribution in octopus studies, oncoming research ought to address the effect of such factors in order to establish the most convenient conditions for being employed as starting substrate. With the same objective, the particular mechanism of changes of element content resulting from cooking and frozen storage ought to be investigated.

## Figures and Tables

**Table 1:** Accuracy control of the analytical procedures for the determination of macro- and microelements in raw and cooked (Column A) and in frozen and cooked frozen (Column B) octopus samples.

Element *	Certified **	Column A	Column B
**Macroelement** (g·kg^−1^)			
Ca	2.01 ± 0.25	1.85 ± 0.09	2.12 ± 0.08
K	11.6 ± 0.4	11.5 ± 0.3	11.5 ± 0.2
Mg	1.03 ± 0.08	1.13 ± 0.05	1.06 ± 0.03
Na	9.20 ± 0.40	9.30 ± 0.24	9.10 ± 0.30
P	6.23 ± 0.24	6.28 ± 0.23	6.35 ± 0.11
S	8.4 ± 0.2	8.5 ± 0.3	8.3 ± 0.1
**Microelement** (mg·kg^−1^)			
As	13.3 ± 0.7	13.4 ± 0.5	13.7 ± 0.4
Ba	0.396 ± 0.023	0.403 ± 0.056	0.409 ± 0.019
Cd	0.148 ± 0.007	0.150 ± 0.004	0.151 ± 0.005
Co	0.063 ± 0.004	0.065 ± 0.002	0.064 ± 0.004
Cu	3.30 ± 0.07	3.39 ± 0.11	3.28 ± 0.03
Fe	113 ± 8	116 ± 3	110 ± 4
Mn	1.06 ± 0.04	0.96 ± 0.03	1.05 ± 0.03
Pb	0.058 ± 0.006	0.054 ± 0.001	0.058 ± 0.001
Sn	0.077 ± 0.008	0.071 ± 0.014	0.073 ± 0.002
Zn	28.7 ± 1.0	29.3 ± 0.7	29.2 ± 0.6

* Data expressed as average value ± standard deviation (*n* = 6). ** DORM-5 from NRC was the certified reference material used [44].

**Table 2:** Content (g·kg^−1^ wet tissue) * of macroelements in different kinds of raw and processed tissues of octopus **.

Macroelement	Raw or Processed	Tissue		
		Arm	Mantle	Viscera
Ca	Raw	0.26 ± 0.01 aA	0.32 ± 0.01 bA	0.36 ± 0.04 bA
	Cooked	0.26 ± 0.02 aA	0.25 ± 0.05 aA	–
	Frozen	0.26 ± 0.02 aA	0.29 ± 0.06 aA	0.33 ± 0.07 aA
	Cooked frozen	0.26 ± 0.03 aA	0.32 ± 0.02 bA	–
K	Raw	3.27 ± 0.06 aB	3.16 ± 0.06 aD	3.22 ± 0.14 aB
	Cooked	1.42 ± 0.20 bA	0.64 ± 0.08 aA	–
	Frozen	3.18 ± 0.27 bB	2.50 ± 0.11 aC	2.41 ± 0.17 aA
	Cooked frozen	1.55 ± 0.16 bA	0.82 ± 0.04 aB	–
Mg	Raw	0.79 ± 0.04 aA	0.92 ± 0.01 bB	0.71 ± 0.04 aB
	Cooked	0.65 ± 0.03 bA	0.50 ± 0.06 aA	–
	Frozen	0.67 ± 0.10 aA	0.91 ± 0.09 bB	0.60 ± 0.06 aA
	Cooked frozen	0.57 ± 0.06 aA	0.56 ± 0.01 aA	–
Na	Raw	5.50 ± 0.14 bC	6.41 ± 0.13 cC	4.57 ± 0.34 aA
	Cooked	3.30 ± 0.09 bB	2.18 ± 0.30 aA	–
	Frozen	5.10 ± 0.46 abC	5.59 ± 0.12 bB	4.37 ± 0.70 aA
	Cooked frozen	2.90 ± 0.16 bA	2.56 ± 0.03 aA	–
P	Raw	2.11 ± 0.06 aB	2.12 ± 0.07 aC	2.83 ± 0.19 bB
	Cooked	1.79 ± 0.08 bA	1.31 ± 0.06 aA	–
	Frozen	2.21 ± 0.26 aB	2.21 ± 0.35 aC	1.97 ± 0.09 aA
	Cooked frozen	1.55 ± 0.16 aA	1.66 ± 0.13 aB	–
S	Raw	6.02 ± 0.04 bB	4.89 ± 0.17 aC	5.97 ± 0.04 bB
	Cooked	4.27 ± 0.37 bA	2.70 ± 0.15 aA	–
	Frozen	6.01 ± 0.22 bB	3.94 ± 0.48 aB	3.98 ± 0.22 aA
	Cooked frozen	4.78 ± 0.45 bA	2.94 ± 0.09 aA	–

* Data expressed as average value ± standard deviation. In each value, data corresponding to three size groups have been pooled together (*n* = 9). ** In each row, different lowercase letters indicate significant differences (*p* < 0.05) among tissues; in each column and for each macroelement, different capital letters indicate significant differences (*p* < 0.05) as a result of processing.

**Table 3:** Content (mg·kg^−1^ wet tissue) * of microelements (As, Cu, Fe, and Zn) in different kinds of raw and processed tissues of octopus **.

Microelement	Raw or Processed	Tissue		
		Arm	Mantle	Viscera
As	Raw	16.5 ± 4.2 aB	12.5 ± 2.3 aC	13.9 ± 3.1 aB
	Cooked	8.03 ± 0.86 bA	4.21 ± 0.66 aA	–
	Frozen	16.2 ± 6.3 bAB	13.4 ± 2.7 bC	7.45 ± 0.63 aA
	Cooked frozen	8.39 ± 1.72 aA	6.01 ± 0.83 aB	–
Cu	Raw	4.09 ± 0.53 aA	7.12 ± 2.77 aA	87 ± 36 bA
	Cooked	11.04 ± 2.52 aB	18.86 ± 2.83 bB	–
	Frozen	5.19 ± 0.64 aA	9.88 ± 3.22 bA	65.7 ± 21.4 cA
	Cooked frozen	14.03 ± 4.48 aB	10.76 ± 2.52 aA	–
Fe	Raw	2.59 ± 0.51 aB	2.74 ± 0.15 aA	157 ± 38 bB
	Cooked	3.19 ± 1.02 aB	3.23 ± 0.39 aB	–
	Frozen	2.22 ± 0.06 aB	5.05 ± 1.96 bC	94.3 ± 41.6 cA
	Cooked frozen	1.25 ± 0.07 aA	3.23 ± 0.22 bB	–
Zn	Raw	15.9 ± 1.0 bA	12.2 ± 0.4 aA	117 ± 4 cB
	Cooked	25.9 ± 3.4 aB	29.4 ± 2.4 aC	–
	Frozen	16.1 ± 0.3 aA	17.4 ± 3.1 aB	112.5 ± 19.5 bA
	Cooked frozen	29.1 ± 5.1 aB	29.9 ± 1.2 aC	–

* Data expressed as average value ± standard deviation. In each value, data corresponding to three size groups have been pooled together (*n* = 9). ** In each row, different lowercase letters indicate significant differences (*p* < 0.05) among tissues; in each column and for each microelement, different capital letters indicate significant differences (*p* < 0.05) as a result of processing.

**Table 4:** Content (mg·kg^−1^ wet tissue) * of microelements (Ba, Cd, Co, Mn, Pb, and Sn) in different kinds of raw and processed tissues of octopus **.

Microelement	Raw or Processed	Tissue		
		Arm	Mantle	Viscera
Ba	Raw	0.024 ± 0.003 bA	0.015 ± 0.003 aA	0.080 ± 0.015 cB
	Cooked	0.024 ± 0.004 aA	0.034 ± 0.004 bB	–
	Frozen	0.022 ± 0.002 aA	0.041 ± 0.004 bB	0.046 ± 0.002 bA
	Cooked frozen	0.024 ± 0.004 aA	0.037 ± 0.007 bB	–
Cd	Raw	0.008 ± 0.001 aA	0.009 ± 0.001 aA	8.78 ± 4.25 bA
	Cooked	0.034 ± 0.021 aB	0.050 ± 0.005 aB	–
	Frozen	0.007 ± 0.001 aA	0.054 ± 0.066 aB	8.30 ± 1.49 bA
	Cooked frozen	0.034 ± 0.012 aB	0.063 ± 0.032 aB	–
Co	Raw	0.008 ± 0.001 aA	0.009 ± 0.003 aA	1.01 ± 0.13 bA
	Cooked	0.010 ± 0.004 aA	0.011 ± 0.001 aA	–
	Frozen	0.007 ± 0.002 aA	0.020 ± 0.017 aA	0.815 ± 0.105 bA
	Cooked frozen	0.006 ± 0.001 aA	0.011 ± 0.002 bA	–
Mn	Raw	0.34 ± 0.04 aA	0.43 ± 0.05 aA	1.54 ± 0.04 bB
	Cooked	0.33 ± 0.07 aA	0.47 ± 0.06 aA	–
	Frozen	0.37 ± 0.02 aA	0.51 ± 0.17 aAB	1.29 ± 0.19 bA
	Cooked frozen	0.34 ± 0.03 aA	0.63 ± 0.05 bB	–
Pb	Raw	0.076 ± 0.021 aA	0.052 ± 0.011 aA	0.989 ± 0.285 bB
	Cooked	0.311 ± 0.141 aB	0.550 ± 0.083 bD	–
	Frozen	0.066 ± 0.011 aA	0.330 ± 0.081 bC	0.303 ± 0.122 bA
	Cooked frozen	0.480 ± 0.170 bB	0.113 ± 0.007 aB	–
Sn	Raw	0.028 ± 0.004 aA	0.044 ± 0.015 abA	0.045 ± 0.004 bB
	Cooked	0.034 ± 0.007 aA	0.030 ± 0.015 aA	–
	Frozen	0.036 ± 0.020 aA	0.039 ± 0.019 aA	0.018 ± 0.004 aA
	Cooked frozen	0.033 ± 0.008 aA	0.028 ± 0.013 aA	–

* Data expressed as average value ± standard deviation. In each value, data corresponding to three size groups have been pooled together (*n* = 9). ** In each row, different lowercase letters indicate significant differences (*p* < 0.05) among tissues; in each column and for each microelement, different capital letters indicate significant differences (*p* < 0.05) as a result of processing.

## Data Availability

The original contributions presented in the study are included in the article, further inquiries can be directed to the corresponding author.
